# Degradation and mechanism analysis of protein macromolecules by functional bacteria in tobacco leaves

**DOI:** 10.3389/fmicb.2024.1416734

**Published:** 2024-07-05

**Authors:** Chuandong Jiang, Decai Kong, Yangyang Li, Jingguo Sun, Zhenguo Chen, Mingfeng Yang, Shoutao Cao, Cunfeng Yu, Zengyu Wang, Jiazhu Jiang, Chengguang Zhu, Nan Zhang, Guangwei Sun, Qiang Zhang

**Affiliations:** ^1^College of Plant Protection, Shandong Agricultural University, Tai’an, China; ^2^Shandong China Tobacco Industry Co., Ltd., Jinan, China; ^3^Hunan Tobacco Research Institute, Changsha, China; ^4^Hubei Provincial Tobacco Research Institute, Wuhan, China

**Keywords:** tobacco, protein degradation, functional bacteria, mechanism analysis, curing

## Abstract

Tobacco, a crop of significant economic importance, was greatly influenced in leaf quality by protein content. However, current processing parameters fail to adequately meet the requirements for protein degradation. Microorganisms possess potential advantages for degrading proteins and enhancing the quality of tobacco leaves, and hold substantial potential in the process of curing. To effectively reduce the protein content in tobacco leaves, thereby improving the quality and safety of the tobacco leaves. In this study, tobacco leaf were used as experimental material. From these, the BSP1 strain capable of effectively degrading proteins was isolated and identified as *Bacillus subtilis* by 16S rDNA analysis. Furthermore, the mechanisms were analyzed by integrating microbiome, transcriptome, and metabolome. Before curing, BSP1 was applied to the surface of tobacco leaves. The results indicated that BSP1 effectively improves the activity of key enzymes and the content of related substances, thereby enhancing protein degradation. Additionally, protein degradation was achieved by regulating the diversity of the microbial community on the surface of the tobacco leaves and the ubiquitin-proteasome pathway. This study provided new strategies for extracting and utilizing functional strains from tobacco leaves, opening new avenues for enhancing the quality of tobacco leaves.

## Highlights

- An effective bacterium capable of breaking down protein was identified through screening.
- The process by which this bacterium decomposes protein was thoroughly investigated.
- This research introduces a fresh approach for isolating functional bacteria from tobacco leaves and understanding their operational methods. It proposes innovative methods for enhancing the quality of tobacco leaves with high efficiency.

## 1 Introduction

Tobacco, especially flue-cured varieties, was a major economic crop. As the leading global producer and consumer, China faces rising demand for high-quality tobacco. However, the quality of Chinese tobacco significantly trails that of internationally recognized high-quality varieties (Hui-Fang et al., 2009). One of the main reasons was that the quality of cured tobacco leaves was adversely impacted by substantial quantities of protein and other macromolecules. Specifically, leaves were made stiff, less oily and supple, and show reduced elasticity and toughness due to high protein content. On the other hand, the combustion performance of tobacco was affected by high protein content, which leads to a burnt taste and irritation, thus diminishing the quality and safety (Chen et al., 2021). Moreover, a reduction in the levels of precursors necessary for aroma compound formation, such as reducing sugars and amino acids involved in the Maillard reaction, can be caused by the incomplete breakdown of starch and protein (Jing and Kitts, 2002). The flavor and aroma of the tobacco leaves were further compromised by this. Additionally, tobacco quality was improved by proper protein management, which reduces cigarette irritation and off-flavors, enhances the aroma of tobacco leaves, and improves the smoking experience (Fu et al., 2020). During the curing process, proteins in tobacco leaves were broken down by endogenous proteases, resulting in the production of small molecules like free amino acids and short peptides. These molecules then underwent the Maillard reaction with sugar molecules, leading to the generation of numerous aroma compounds. Following modulation, the internal chemical composition of the tobacco leaves became more balanced, significantly improving their quality (Jing and Kitts, 2002). Hence, the reduction of protein content in tobacco leaves was crucial for enhancing tobacco quality and its applicability. The vital role played by the tobacco modulation process in highlighting the quality attributes of tobacco leaves was recognized. This was also the main period during which large molecules such as proteins were extensively degraded, and aroma compounds were synthesized. The degradation of proteins during this stage was identified as an important factor in improving the quality of tobacco leaves (Li et al., 2019). Although researchers have made many efforts, it was difficult to achieve the necessary protein degradation through process parameter adjustments alone.

The critical importance of microorganisms residing on tobacco leaf surfaces in enhancing tobacco quality throughout the curing stage has been highlighted by recent discoveries. The essential functions of these microbial communities in advancing the fermentation of tobacco have been recognized as significantly improving both the quality and scent of the tobacco (Hu et al., 2021; Wu et al., 2022; Zheng et al., 2022). Several experiments had been conducted to confirm this; for example, the application of a combination of *Bacillus amylus* and *Bacillus Gauciformii* to tobacco leaves was found to result in a decrease in protein content and improvements in the aroma of the leaves (Wu et al., 2021). Aroma development in cured tobacco leaves can be facilitated by the isolation and application of thermophilic bacteria, such as *B. subtilis*, from fermented leaves (Dai et al., 2020). *B. subtilis* strains capable of degrading proteins were identified and isolated from high-quality tobacco leaves during the fermentation process. When this strain was applied to lower-grade tobacco leaves, their quality was significantly enhanced (Ma et al., 2023). During the fermentation process, an improvement in the quality of tobacco fermentation can be effectively achieved by an increase in the abundance of *Pseudomonas* and a decrease in the abundance of *Burkholderia* and *Methylobacterium* (Li et al., 2020). However, several obstacles were encountered in microbial protein degradation in tobacco leaves, including low technological development and limited scalability, which impede efficient protein identification. Most microbial growth and degradation activities were deterred by high temperatures during the curing process. Furthermore, microbial function was inhibited by tobacco metabolites such as nicotine and nicotinic acid. Due to these challenges, scant research exists on microbes that degrade protein during the curing process. Therefore, it was crucial to identify and study functional bacteria capable of degrading protein under these conditions.

In this study, for the first time, functional bacterial strains adept at degrading proteins during tobacco curing were screened from tobacco leaves, and *B. subtilis* was identified through 16S rRNA amplification and phylogenetic analysis. When applied to tobacco leaves during curing, the breakdown of macromolecules such as proteins was enhanced by the BSP1 strain. By exploring the degradation mechanism using metagenomics, transcriptomics, and metabolomics, new avenues for extracting protein-degrading bacteria and enhancing tobacco curing quality were opened by the study.

## 2 Methodology and materials

### 2.1 Materials and location

The tobacco variety Yunyan 87, provided by the Hubei Tobacco Science Institute, was employed in the research. The screening for functional microorganisms utilizing leaves collected from tobacco was conducted at the Baiyangba Tobacco Station in Lichuan City, within Enshi Prefecture, Hubei Province. Freshly harvested, medium-matured tobacco leaves, treated according to standardized harvesting and curing methods for consistency, were the focus of the research. The curing process was undergone by these tobacco leaves in a curing barn located at the Baiyangba experimental base in Enshi City.

### 2.2 Isolation and screening of functional bacteria in tobacco

Ten grams of tobacco leaves were mixed with 90 mL of sterile water in a sterilized flask and shaken at 37°C and 230 rpm. Following a 4-h incubation period, the solution was serially diluted and then spread onto Luria-Bertani (LB) agar plates. These plates were then incubated at 37°C for 24 h. Isolated colonies were purified, stored at −80°C in 15% glycerol, and reactivated in protein culture medium. After 24 h of cultivation, the dried bacterial solution was placed in an incubator at 37°C for 24 h, and the formation of a transparent circle was observed.

The activated bacterial solution was diluted and introduced onto skim milk agar plates, which were then inverted and incubated at 37°C for 24 h. When a specific size was reached, iodine solution was applied to the edges of the colony. Following incubation, the diameters of the clear zones (H) surrounding the bacterial colonies and the diameters of the bacterial colonies themselves (C) were measured, and the H/C ratio was calculated. Strains with larger hydrolysis zones underwent further analysis for the size of the protein transparent zone and protease activity, which indicated their protein degradation capability.

### 2.3 Genome extraction, amplification, and sequencing

#### 2.3.1 Amplification of the 16S rRNA gene

The process of genome extraction, amplification, and sequencing involved the use of 16S rRNA universal primers, specifically 27F and 1492R (Coenye et al., 1999). The sequences for these primers were 27F (5’-AGAGTTTGATCCTGGCTCAG-3′) and 1492R (5’-GGTTACCTTGTTACGACTT-3′). The Polymerase Chain Reaction (PCR) protocol started with an initial denaturation at 94°C for 5 min. Thirty cycles consisting of denaturation at 94°C for 1 min, annealing at 50°C for 1 min, and extension at 72°C for 3 min and 15 s were followed by a final extension phase at 72°C for 10 min. High-quality PCR products were then purified and sent to Wuqing Kechuangxin Biotechnology Co., Ltd. for sequencing analysis.

#### 2.3.2 Construct phylogenetic tree analysis

Following the sequencing of the amplified gene, a homology comparison analysis was conducted using the National Center for Biotechnology Information (NCBI) platform. A phylogenetic tree was then constructed using MEGA4.0 software based on the results of this analysis. The evaluation of genetic relationships and evolutionary distances between the identified sequences and known sequences in the database was facilitated by this process (Kumar et al., 2004).

### 2.4 Application of functional bacteria and determination of samples

An equal volume of distilled water was used to dilute the bacterial fermentation solution, achieving a 1:1 ratio. This mixture was then evenly sprayed onto both sides of the tobacco leaves, ensuring uniform coating without causing excess runoff or dripping.

#### 2.4.1 Determination of enzyme activity and related substances during curing

Tobacco leaves treated with both water and a specific bacterial fermentation solution were processed in a curing barn using a staged three-phase method. During this process, leaf samples were collected at various temperatures: 38°C, 40°C, 42°C, 44°C, and 46°C, as well as post-curing, once the leaves had returned to room temperature. The activity levels of enzymes and compounds such as peroxidase (POD) (Doerge et al., 1997), superoxide dismutase (SOD) (Zhou and Prognon, 2006), polyphenol oxidase (PPO) (Houlong et al., 2017), nitrate reductase (NR) (Wany et al., 2020), malondialdehyde (MDA) (Davey et al., 2005), and protein concentration (Ma et al., 2023) were measured.

#### 2.4.2 Extraction and determination of microbiome, transcriptome, and metabolome analysis samples

Tobacco leaves that were subjected to functional bacterial treatment underwent an intensive three-stage curing process in a barn. Samples collected at 40°C were promptly transported on ice to the laboratory and subsequently stored at −80°C for further omics analysis. These procedures were carried out at Beijing Novogene Biotechnology Co., Ltd., where good reproducibility and a stable analysis system were confirmed by quality control (QC). Data analysis employing multivariate statistical techniques was carried out using SIMCA-P11 software to interpret the findings.

### 2.5 Multi-omics analysis

#### 2.5.1 Microbiome-related analysis

The complexity and variety of bacterial communities in different treatments were evaluated and compared in this research by employing Operational Taxonomic Units (OTUs), along with Chao1, Simpson, and Shannon indices, to measure biodiversity. Bacterial richness in tobacco leaves treated differently was assessed through the analysis of OTUs, Chao1, and Shannon indices in the collected samples (Nossa et al., 2010). Furthermore, the extent of bacterial diversity was explored through the calculation of species coverage indicated by OTUs, which provided deeper insights into the microbial ecosystem present on the tobacco leaves.

#### 2.5.2 Transcriptome analysis results

The analysis of genes expressed differently was performed with DESeq2, setting the parameters to a fold change of |Log2| ≥ 1 and a significance level of *p* < 0.05 (Love et al., 2014). Annotations from KEGG and GO databases were visualized using the ggplot2 package, while enrichment analysis utilized a clustering analysis tool (Huerta-Cepas et al., 2017), Data visualization leveraged the R programming language and TBtools for comprehensive insights (Yu et al., 2012; Chen et al., 2020). Furthermore, the Weighted Gene Co-expression Network Analysis (WGCNA) package in R (Version 3.5.0) was employed to process gene co-expression networks, enabling the identification and visual analysis of key genes within the network (Diboun et al., 2006).

#### 2.5.3 Differential metabolite screening and metabolic pathway analysis

In this study, orthogonal partial least squares discriminant analysis (OPLS-DA) was utilized to identify differential metabolites (DEMs), alongside the creation of a PLS-DA model for these metabolites. The model’s ability to predict was verified using R2 and Q2 values derived from permutation testing. DEMs were selected based on having a Variable Importance in Projection (VIP) greater than 1.0 and a *p*-value of less than 0.05, ensuring a robust analysis of metabolite differences (Usadel et al., 2009). In this study, the count of up-regulated and down-regulated differential metabolites (DEMs) was determined, and the expression patterns of the ten DEMs with the highest Variable Importance in Projection (VIP) scores were closely examined. Additionally, the identified DEMs underwent functional and categorical annotation using the Human Metabolome Database (HMDB). To visually depict the variations in expression patterns across the metabolites, a clustering heatmap was created, facilitating an intuitive understanding of the data.

### 2.6 Data analysis

This research involved the processing of all statistical and analytical data through the use of multiple software tools, such as the DPS data processing system, IBM SPSS Statistics, Origin 2019b, R studio, and Excel 2016.

## 3 Results

### 3.1 Screening of protein-degrading functional bacteria

**Figure 1:**
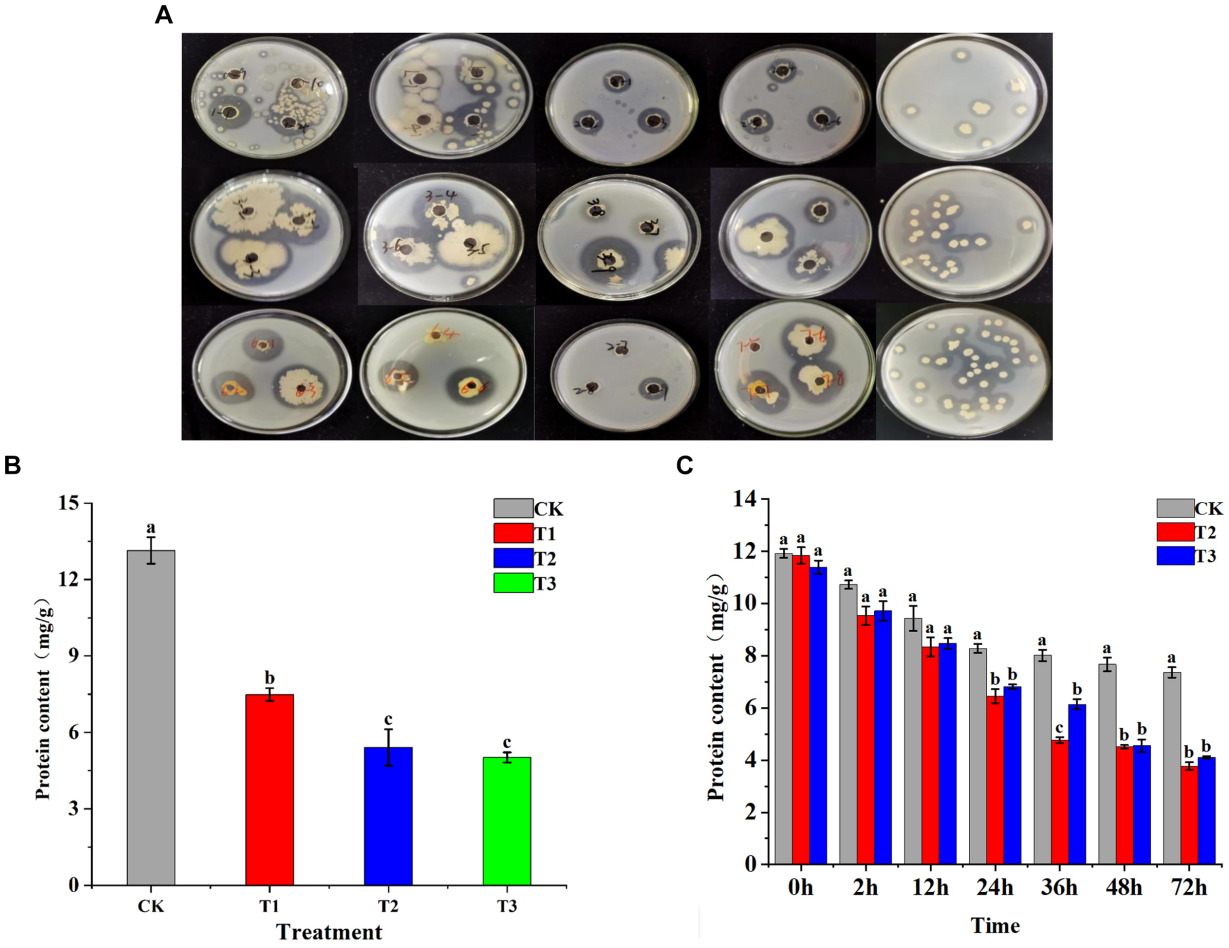
Illustrates the screening process for functional bacteria and their impact on the protein content of tobacco leaves. **(A)** Shows the initial screening of bacteria capable of degrading protein in tobacco leaves, employing selective culture media for identification. **(B)** Depicts the protein degradation activity in fresh tobacco leaves by strains T1, T2, and T3 at the 24-h mark. **(C)** Extends this observation of protein degradation by strains T2 and T3 over multiple time points: 2 h, 12 h, 24 h, 48 h, and 72 h, providing a detailed view of their activity over time.

Using the dilution plating technique, sixty bacterial strains along with three fungal strains were isolated from surfaces of tobacco leaves. Among them, 37 strains demonstrated proteolytic activity, forming clear zones on skim milk agar plates (Figure 1A). Nineteen strains with larger zones of hydrolysis were selected for detailed investigation. After 24 h of shaking incubation, 14 of these strains demonstrated an H/C (hydrolysis/culture) ratio greater than 1.5. Strains 4, 5, and 18 notably exhibited the highest amylase activity, measuring 66.96 ± 5.50 U/mL, 71.05 ± 0.64 U/mL, and 74.4 ± 5.2 U/mL, respectively (Supplementary Table S1). Fermentation solutions designated as T1, T2, and T3 were formulated from these strains and sprayed uniformly on tobacco leaves, with sterile water used as CK. The protein content of the tobacco leaves was assessed 24 h after the application (Figure 1B) In all experimental groups, a significantly lower protein content compared to CK was observed, indicating a substantial impact of the functional bacteria on protein degradation. The effect was notably more pronounced in the T2 and T3 treatments, demonstrating the superior capacity for protein breakdown of strains T2 and T3. Strains T2 and T3 were evenly applied to both sides of the tobacco leaves, together with an equivalent volume of water, to assess the temporal dynamics of their action. This approach was used to determine the optimal duration of activity for these strains (Figure 1C). After 36 h, the efficacy of bacterial strains T2 and T3 in degrading proteins in tobacco leaves was assessed. Initial results revealed no significant difference in protein degradation between T2, T3, and CK at the 24-h mark. However, the effectiveness of these bacterial strains in breaking down proteins became more apparent over time. The degradation rates by T2 were 22.1% at 24 h and 48.78% at 72 h, showcasing T2’s superior and more consistent performance in protein degradation compared to T3 (Supplementary Table S2). Consequently, T2 was selected as the more effective strain for protein degradation purposes.

### 3.2 Functional identification of bacteria

**Figure 2:**
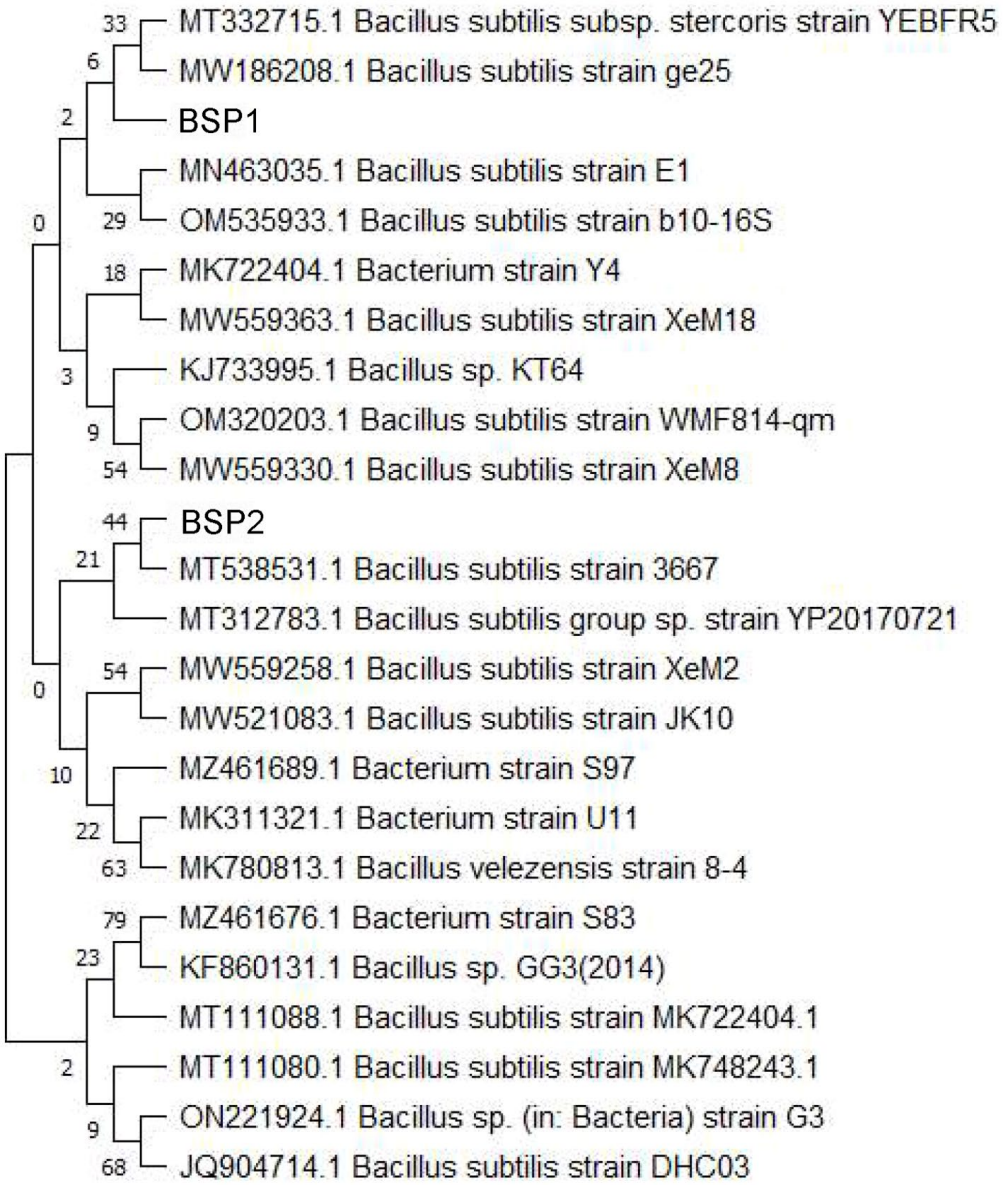
Phylogenetic tree based on 16S rDNA sequences.

Strain T2 was identified following the amplification, purification, and sequencing of its 16S rDNA. A homology comparison with nucleic acid sequences in the GenBank database was conducted to ascertain its similarity level. This analysis revealed that *Bacillus subtilis* (MT111088) showed the highest similarity to T2. Subsequently, a phylogenetic tree incorporating T2 and closely related strains was constructed using the neighbor-joining method (Figure 2). Strain T2 was found to have the closest evolutionary relationship with *Bacillus subtilis* MW186208. From these findings, strain T2 was confirmed to be *Bacillus subtilis* and has been designated as BSP1.

### 3.3 The impact of functional bacterial treatment on physiological parameters of cured tobacco leaves

**Figure 3:**
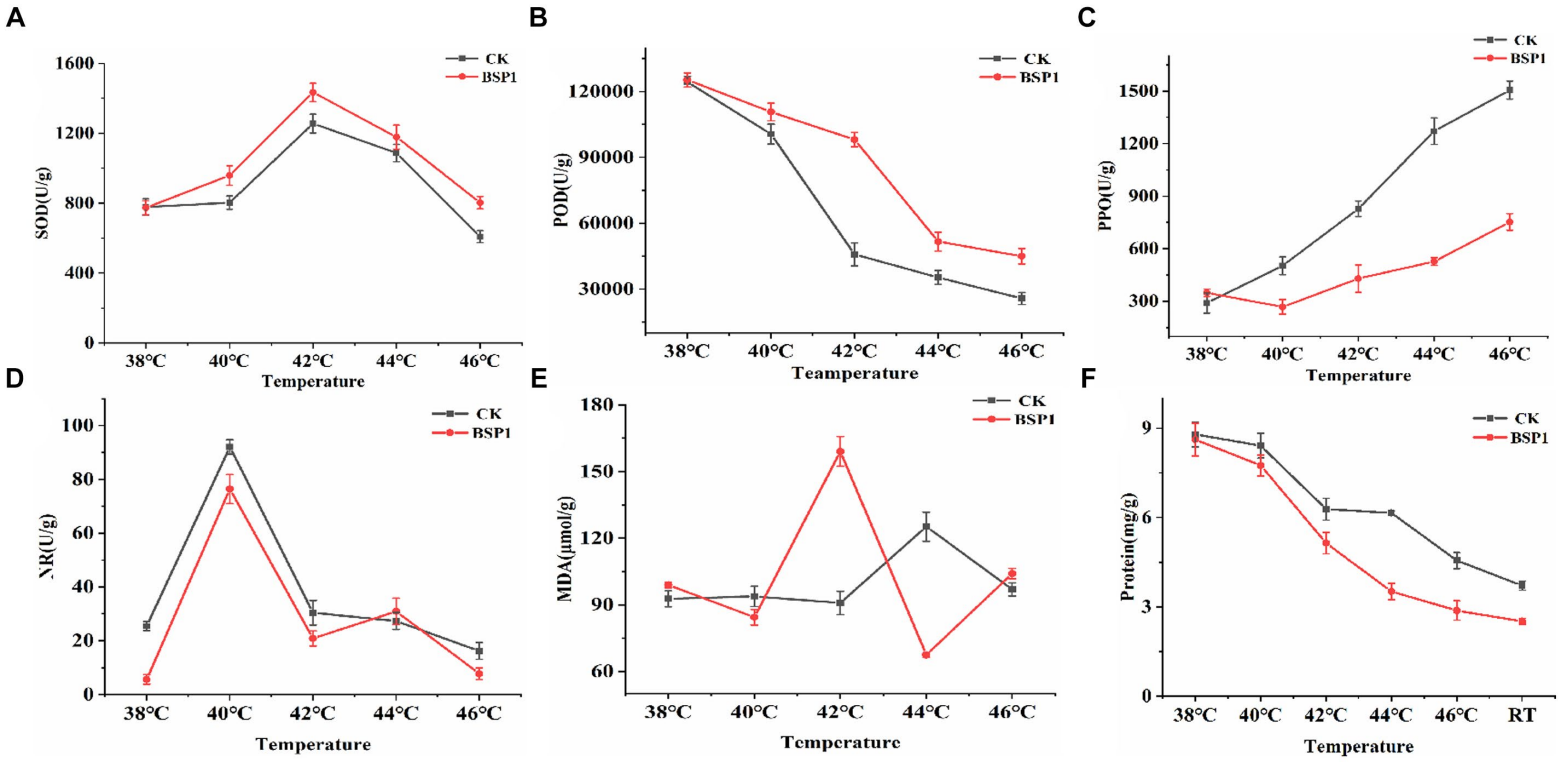
Changes in physiological indicators of tobacco leaves in different treatment groups during: **(A)** SOD, **(B)** POD, **(C)** PPO, **(D)** NR, **(E)** MDA, and **(F)** Protein content. *RT: cool to room temperature after curing.

SOD, POD, MDA, PPO, NR were important factors that influence protein degradation of tobacco leaves during curing process (Hua-Wu et al., 2012; Meng et al., 2022). Therefore, the changes in Enzymes activity were measured (SOD, POD, PPO, NR) at temperatures of 38°C, 40°C, 42°C, 44°C, and 46°C during curing process (Figures 3A–D). The enzyme activity of SOD and POD was effectively improved by BSP1 during the curing process, leading to the promotion of degradation and transformation of tobacco leaves constituents and the indirect influence on the quality of tobacco leaves. Concurrently, PPO activity decreased and changed at a slower rate, indicating that the tobacco leaves were less susceptible to undergoing browning reactions. Moreover, the NR activity decreased, indicating that a role was played by BSP1 in reducing the speed of nitrogen metabolism, reducing nitrogen assimilation during curing process. Secondly, the content of related substances during the curing process of tobacco leaves was measured (Figure 3E). The results indicated that the significant reduced in the MDA content was observed at 42°C. Curing speed of tobacco leaves was accelerated, and an enhancement in their resistance to environmental stress (Chen et al., 2019; Li et al., 2019; Yang et al., 2021). Finally, tobacco leaves were collected and the protein content were measured at 38°C, 40°C, 42°C, 44°C, 46°C as well as Cool to room temperature after curing. Subsequently, all the samples were crushed, and their protein content was determined. After applying BSP1, a continuous and rapid decrease in protein content was observed at temperatures ranging from 40°C to 46°C, but CK was changed gently between 42°C and 44°C. Compared to the CK, the degradation rate of the tobacco leaves treated with BSP1 increased by 36.90%. At the end of the curing process, a 29% increase in protein degradation rate after the application of BSP1 (Figure 3F). This resulted that the application of the BSP1 strain can result in a lengthened degradation time and a faster degradation rate of tobacco leaf proteins during the curing process, resulting in more efficient degradation of protein content in tobacco leaves.

### 3.4 Analysis of the impact of applying functional bacteria on the diversity of bacterial and communities in tobacco leaves

**Figure 4:**
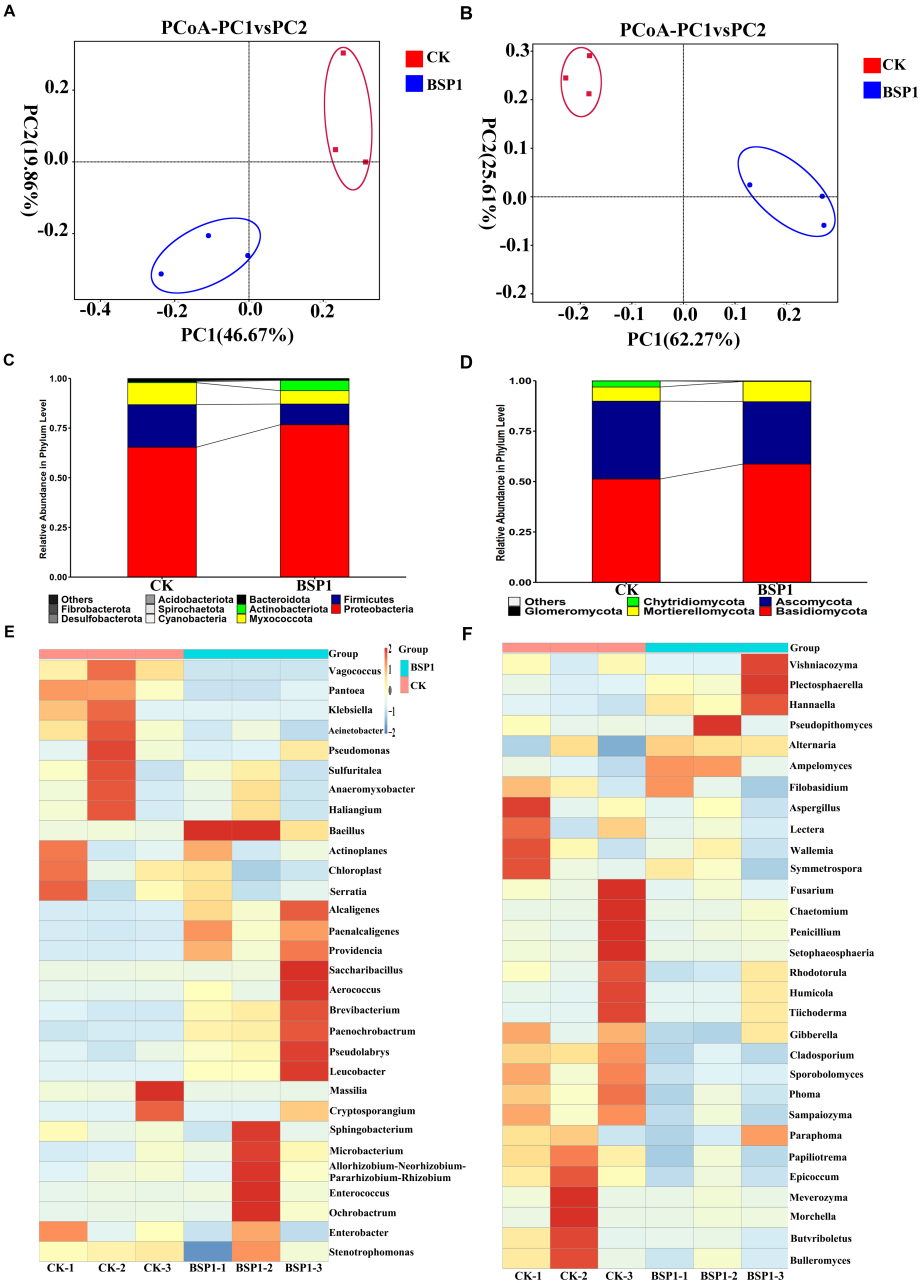
The microbiome analysis under various treatments. **(A)** The PCoA for bacterial; **(B)** the PCoA for fungal; **(C)** phyla analysis of bacteria; **(D)** phyla analysis of fungal; **(E)** genus analysis of bacteria; and **(F)** genus analysis of fungal.

In terms of bacterial diversity: The analysis of bacterial community abundance was conducted using OTUs, Chao1, Shannon and Simpson index (Supplementary Table S3). This resulted that compare to CK, the BSP1 group displayed higher OTUs, Chao1, and Shannon indices. Notably, the Shannon index of the BSP1 group was significantly greater than that of the CK, indicating a statistically significant difference (*p* < 0.05). Furthermore, the Simpson index for the BSP1 group was significantly lower compared to the CK (*p* < 0.05), suggesting a higher biodiversity within the BSP1 group relative to the CK. As a result, it was determined that the species diversity and community abundance on tobacco leaves were significantly higher in the BSP1 group than in the CK. Additionally, the coverage of OTU species exceeded 0.99, indicating extensive library coverage. This high level of coverage suggests that the diversity of bacterial species present in the samples was well-represented, offering a comprehensive view of the microbial ecosystem associated with the tobacco leaves. Furthermore, Principal Coordinates Analysis (PCoA) show that CK group and BSP1 group have obvious separation in the distribution of sample points. This separation indicates that the application of BSP1 had a significant impact on the bacterial community structure of the tobacco leaves, altering the microbial ecosystem in a way that was distinct from the untreated control group (Figure 4A). At the phylum level, the bacterial composition was primarily dominated by the phylum *Proteobacteria*, which accounted for over 55% of all bacterial taxa in both the CK and BSP1 group. After application of the BSP1, the abundance of *Actinobacteria* in tobacco leaves was higher compared to the CK. In contrast, the phylum *Firmicutes*, *Myxococcota* and *Bacteroidetes* were less abundant in the BSP1 group (Figure 4C).

The heatmap analysis was performed on the top 30 genera, using hierarchical clustering, to determine the distinct composition of bacterial community structure (Figure 4E). The application of the BSP1 strain significantly increased the abundance of *Bacillus*, *Kurthia*, *Alcaligenes*, *Staphylococcus*, *Corynebacterium*, *Chromobacterium*, *Pseudomonas*, *Sphingomonas*, *Microbacterium*, *Enterococcus*, and *Flavobacterium* in tobacco leaves. In contrast, the BSP1 group significantly decreased the abundance of *Acinetobacter*, *Halobacillus*, *Salinibacterium*, *Serratia*, *Pseudomonas*, *Thiothrix*, *Anaerobacter*, *Myxococcus*, *Escherichia*, *Cryptococcus*, *Klebsiella*, and *Pantoea*. These results indicate that the certain impact on the bacterial community structure was observed as a result of the application of the BSP1 strain on tobacco leaves. Additionally, the genus *Bacillus* was known for its highly effective protease activity, which can efficiently promote protein degradation and enzymatic hydrolysis (Spencer et al., 2004; Kristensen et al., 2021). The results indicate that the bacterial community was significantly enriched through the application of BSP1, particularly the genus *Bacillus*, which possesses high specific activity of proteolysis with efficient protease system and can effectively degrade proteins in plant.

In terms of fungal diversity: The analysis of bacterial community abundance was conducted using OTUs, Chao1, and Shannon indices (Supplementary Table S4). This resulted that the BSP1 group had significantly lower OTU and Chao1 indices compared to the CK (*p* < 0.05). The Simpson index was found to be lower in the BSP1 group, while the Shannon index was observed to be higher than that in the CK, but the differences were not determined to be statistically significant. This resulted that the abundance of fungal communities was reduced by the BSP1, but the species diversity of tobacco leaves was increased. It resulted that an important role in altering the fungal diversity in tobacco leaves and influencing the species diversity of fungal communities was played by BSP1. Additionally, the OTU species coverage was calculated to be greater than 0.99, indicating high library coverage, which accurately reflects the diversity of fungal species.

PCoA analysis demonstrated a clear divergence in sample point distribution between the CK and BSP1 groups. This distinct separation underscores the significant impact of applying the BSP1 strain on the fungal community structure within tobacco leaves (Figure 4B). At the phylum level, the fungal composition was primarily dominated by the phylum *Basidiomycota*, which accounted for over 52% of all fungal taxa in both the CK and BSP1 groups. After the application of BSP1, the abundance of *Ascomycota* and *Zygomycota* in tobacco leaves was higher compared to the CK group. In contrast, the phylum *Glomeromycota* was more abundant in the CK (Figure 4D). A heatmap analysis was performed on the top 30 genera, using hierarchical clustering, to determine the distinct composition of fungal community structure. In the heatmap analysis of the fungal community (Figure 4F), the relative abundance of genera such as *Penicillium*, *Rhizopus*, *Fusarium*, *Cladosporium*, *Aspergillus*, *Trichoderma*, *Candida*, *Mortierella*, *Mucor*, *Alternaria*, *Stachybotrys*, *Issatchenkia*, *Stemphylium*, *Fusarium*, *Morchella*, *Amanita*, *Bullera*, and *Melampsora* was significantly lower in the BSP1 group compared to the CK group. On the other hand, the relative abundance of genera such as *Sporobolomyces*, *Cryptococcus*, *Hannaella*, *Sphingomonas*, and *Erysiphe* was higher in the BSP1 group than in the CK. Indeed, the genus *Penicillium* was known for its abundant protease activity and played a significant role in protein degradation and nitrogen metabolism (Woodward and Bartel, 2005). The genus Aspergillus, including species like *Aspergillus*, was an important producer of proteases. It was widely used in food fermentation and industrial production of proteases (Dharmasiri and Estelle, 2004; Behera et al., 2019). The findings indicate that the BSP1 strain significantly enriched the communities of fungi capable of degrading protein, potentially leading to an accelerated rate of protein degradation in tobacco leaves (Sriranganadane et al., 2010). This enhancement of protein-degrading fungal communities suggests a targeted effect of BSP1 on promoting specific microbial interactions that benefit the decomposition process in the leaf substrate.

### 3.5 Transcriptomic analysis of tobacco leaves after the application of BSP1

**Figure 5:**
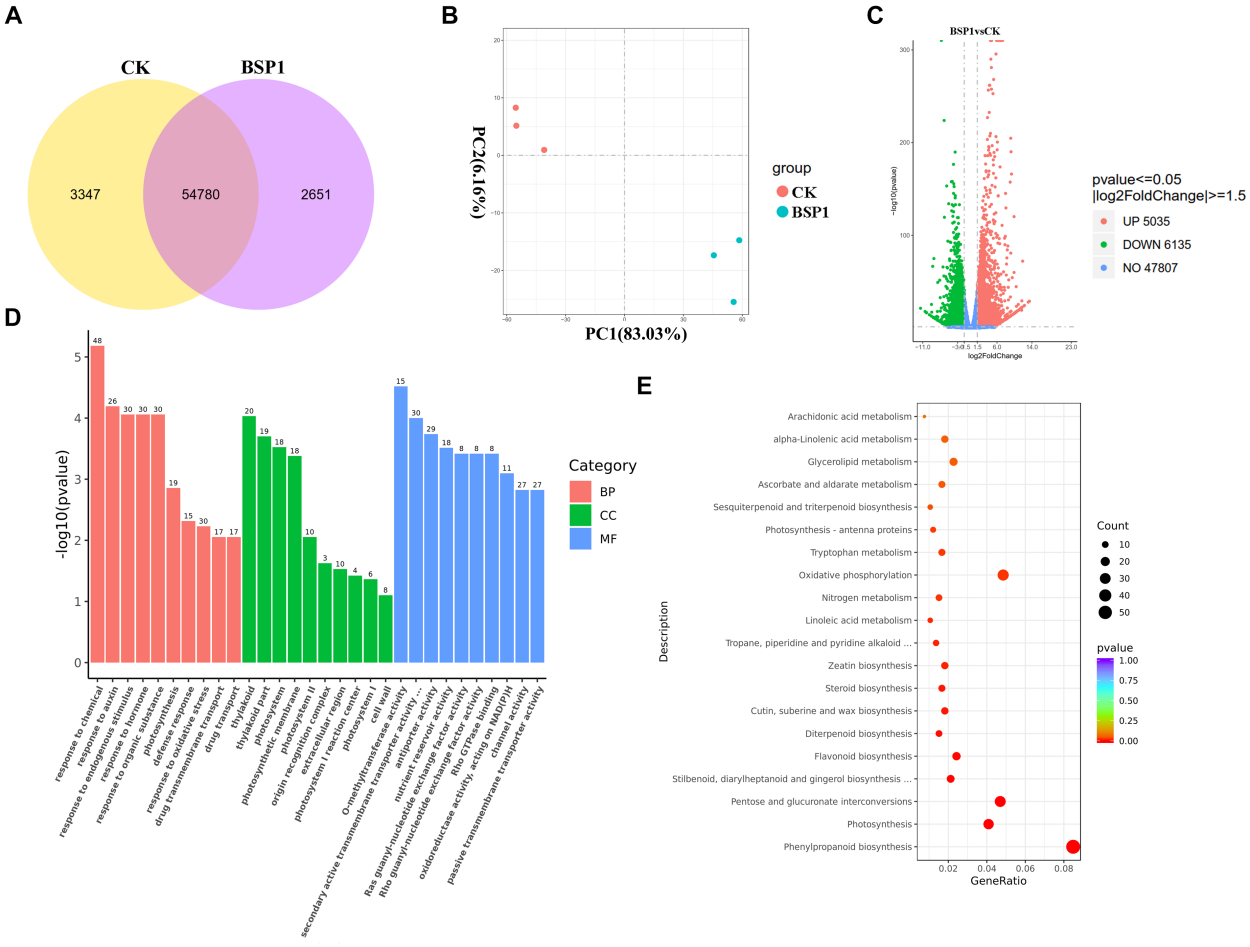
The examination of gene expression patterns within the tobacco leaf transcriptome. **(A)** Analysis of Venn diagram co-expression; **(B)** analysis of PCA principal component; **(C)** a volcano plot visualizing of DEGs; **(D)** GO functional analysis of DEGs in different treatments with molecular functions in red, biological processes in green, and cellular components in blue; **(E)** KEGG enrichment analysis of DEGs.

To investigate the mechanism of action of BSP1 accelerating protein degradation in tobacco leaves, PCA was performed on the RNA-seq samples. Distinct clustering and separation between the BSP1 and the CK group were exhibited by the samples, indicating good specificity (Figure 5B). This resulted significant differences in gene transcription levels between tobacco leaves treated with BSP1 and the CK group. The analysis showed that there were 54,780 genes expressed in both the BSP1 and CK group. Specifically, there were 58,127 expressed genes in the BSP1 group, while 57,431 expressed genes were observed in the CK. Furthermore, there were 3,347 genes specifically expressed in the BSP1 group and 2,651 genes specifically expressed in the CK (Figure 5A). Gene selection on all differentially expressed genes was performed by setting thresholds (VIP > 1.0, FC > 1.5, *p* < 0.05). This resulted that the expression of 11,170 genes was significantly altered by the BSP1 group compared to the CK. Among these genes, 5,035 were upregulated, and 6,135 were downregulated. Interestingly, there were more downregulated genes than upregulated genes after the application of BSP1 (Figure 5C). These results suggested that multiple physiological processes might be enhanced under the action of functional bacteria.

To explore the roles of the 11,170 DEGs, a GO enrichment analysis was conducted (Figure 5D). This analysis demonstrated that the DEGs were significantly grouped into three main categories: molecular functions, biological processes, and cellular components. Within the molecular functions, the methylation process of methyl transferase activity, secondary active transmembrane transporter activity, antiporter activity, and nutrient reservoir metabolic process were observed in the following four components among the differentially expressed genes. Within the biological process functions, the enriched components include metabolic processes related to auxin response, metabolic processes related to endogenous stimulus response, metabolic processes related to hormone response, and metabolic processes related to organic substance response. In the cellular component category, the enrichment was mainly observed in components such as plastids, photosynthetic membranes, and photosystems.

Furthermore, an analysis focusing on the KEGG pathways was conducted, as illustrated in Figure 5E, to determine the specific metabolic pathways involving the DEGs. This analysis revealed that the DEGs contributed to 115 distinct pathways, among which 111 pathways exhibited notable differences. This indicates a broad involvement of the DEGs in various critical biological processes and metabolic functions. Based on the *p*-value, the top 20 pathways with the most significant enrichment were selected to plot the KEGG enrichment scatter plot. This resulted that the phenylpropane biosynthesis signaling pathway, nitrogen metabolism signaling pathway, phenylalanine metabolism, flavonoid biosynthesis, tryptophan metabolism, steroid biosynthesis and metabolism pathways, and the photosynthesis-antenna protein signaling pathway were enriched with the application of BSP1.

### 3.6 Construction of weight gene co-expression network

**Figure 6:**
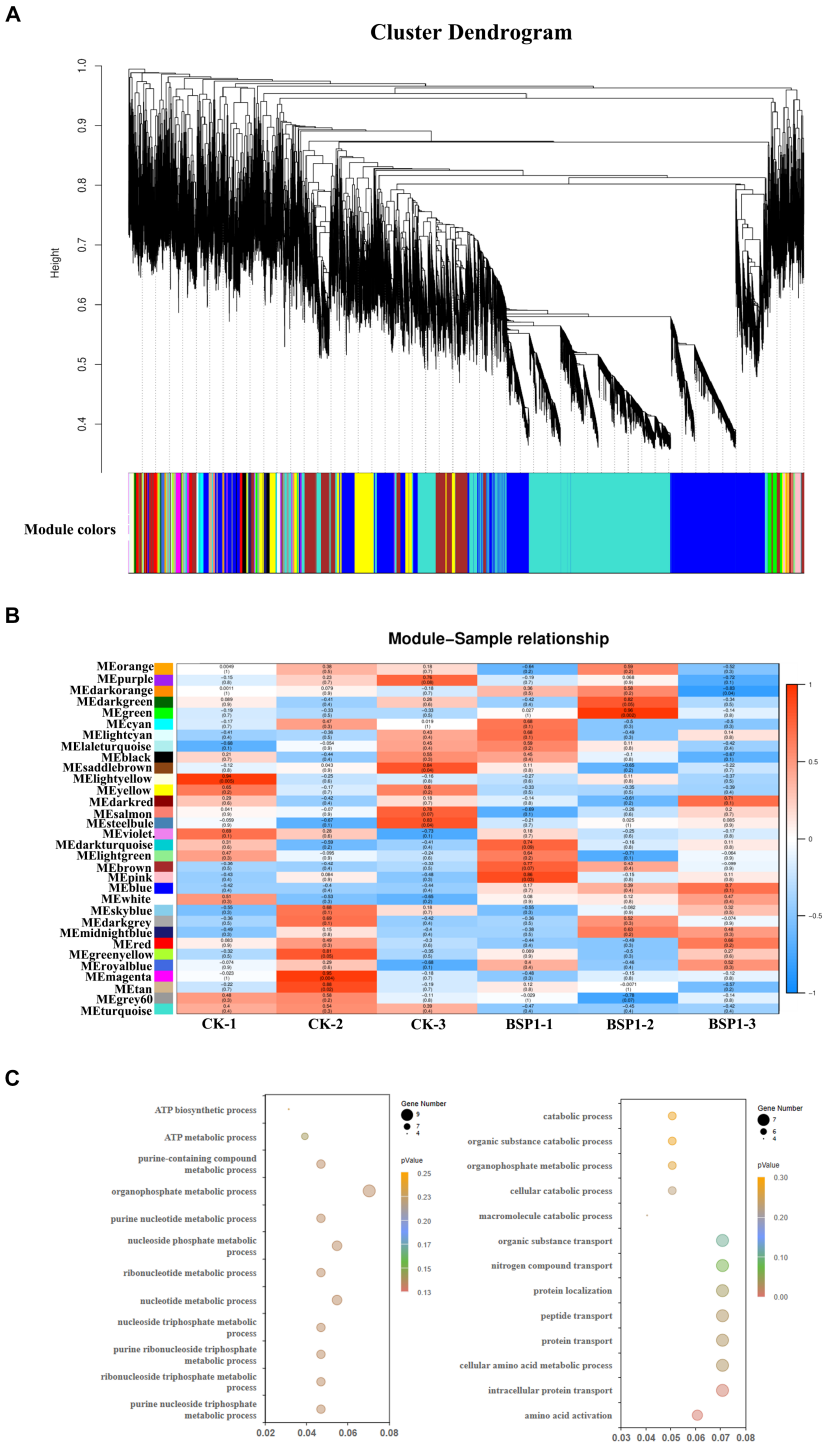
The construction of a weighted gene co-expression network **(A)** Clustering dendrogram of genes and module division. **(B)** An association analysis between the identified modules and tissue types in the context of BSP1 treatment. **(C)** Green and Pink module GO analysis result.

All 35,457 genes were included in an unsigned Weighted Gene Co-expression Network Analysis (WGCNA) to study the co-expression network associated with BSP1. The WGCNA method successfully generated the BSP1 co-expression network, which was organized into 32 distinct modules (Figures 6A,B). Notably, the green and pink modules showed a significant positive correlation with BSP1, containing 686 and 403 genes, respectively. The green module, with a correlation of 0.86, was linked to nucleotide metabolism, while the pink module, correlating at 0.96, was associated with the catabolism of proteins and macromolecules. These modules may reflect tissue-specific biological processes. To assess the accuracy of the co-expression network and the relevance of the tissue-specific module, an analysis was conducted on the module related to protein degradation in tobacco mediated by BSP1.

Analysis of the two tissue-specific modules associated with BSP1 indicated significant enrichment in the biological processes related to nucleotide metabolism and the catabolism of proteins and macromolecules. GO enrichment analysis was performed on these modules, revealing substantial enrichment in processes involved in the decomposition of organic macromolecules, as well as in pyrimidine and purine metabolic pathways (Figure 6C). Specifically, the pink module was significantly enriched in pathways related to the degradation of organic macromolecules, as evidenced by enrichment in terms such as the catabolic process (GO:0009056), macromolecule catabolic process (GO:0009057), and organic substance catabolic process (GO:1901575). Conversely, the green module primarily focused on the metabolism of pyrimidines and purines, as well as processes related to ATP metabolism. This was demonstrated through significant enrichment in areas like nucleotide metabolic process (GO:0009117), purine nucleotide metabolic process (GO:0006163), and ATP biosynthetic process (GO:0006754).

### 3.7 Metabolomic analysis of tobacco leaves after the application of BSP1

**Figure 7:**
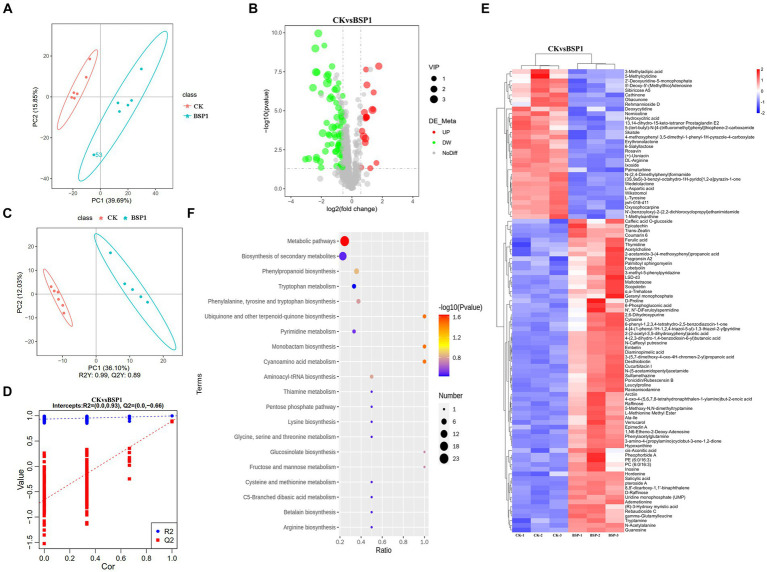
The examination of expression patterns within the tobacco leaf metabolome. **(A)** Analysis of PCA principal component; **(B)** volcano plot of DEMs; **(C,D)** analysis of PLS-DA plot; **(E)** cluster analysis of DEMs. **(F)** KEGG of DEMs.

To further investigate the mechanism of action of BSP1 on tobacco leaves, metabolomic analysis was conducted on the CK and BSP1 groups. A comprehensive analysis identified 99 metabolites showing significant variations, comprising 67 upregulated and 32 downregulated metabolites (Figure 7B). The PCA revealed marked distinctions in the clustering and separation of samples between the BSP1 and CK (Figure 7A), Furthermore, the OPLS-DA model (*p* < 0.05, VIP > 1), aligned with the PCA findings (Figures 7C,D). These findings further elucidate the metabolites of tobacco leaves were significantly impacted by the application of BSP1.

In addition, functional and classification annotations of the identified metabolites were performed using the HMDB (Figure 7E). The results showed that the upregulated differentially expressed metabolites (DEMs) were Amino acid metabolites such as Tryptamine, L-cysteine, tryptophan with the application of BSP1. Conversely, the downregulated DEGs were amino acid metabolites such as Nornicotinic acid, wikstroemia, salvic acid and wedelactone. Subsequently, we conducted KEGG pathway enrichment analysis on the DEMs (Figure 7F) and the pathway of significant enrichment were biosynthesis of amino acids, biosynthesis of secondary metabolites and phenlpropanoid biosynthesis.

### 3.8 Multi-omics association analysis

**Figure 8:**
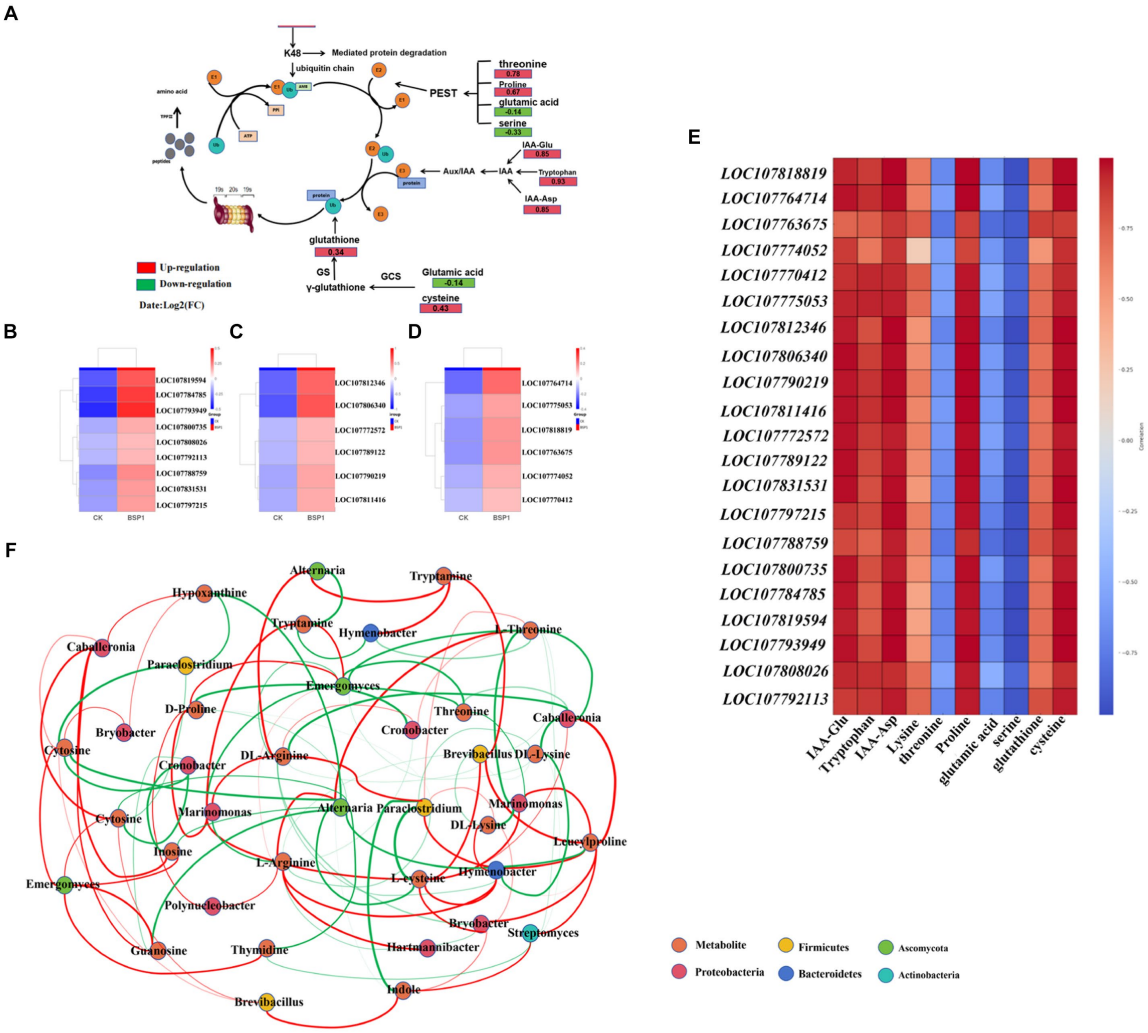
The network analysis **(A)** the analysis of protein metabolism network pathway. **(B,C)** Related genes of amino acid metabolism. **(D)** Related genes of auxin metabolism. **(E)** The analysis of correlation heat map. **(F)** Analysis correlating metagenome and metabolome data: Red lines indicate a positive correlation, while green lines denote a negative correlation. The thickness of each line illustrates the intensity of the correlation (*p* < 0.5).

Further analysis of the joint transcriptome was conducted, aiming to construct metabolic network pathways impacting the functional bacteria influence protein degradation (Figure 8A). Additionally, heat maps illustrating the correlation between the transcriptome and metabolome was created (Figures 8B–E). After the application of BSP1, significant changes were observed in the metabolic levels of amino acids and auxin precursors. The levels of glutathione (0.34), cysteine (0.43), Lysine (0.39), threonine (0.78), Proline (0.67) in the BSP1 group were found to be significantly higher than those in the CK group. Genes associated with amino acid metabolism (*LOC107812346, LOC107806340, LOC107790219, LOC107811416, LOC107772572, LOC107789122, LOC107831531, LOC107797215, LOC107788759, LOC107800735, LOC107784785, LOC107819594, LOC107793949, LOC107808026* and *LOC107792113*) were significantly upregulated. Among them, cysteine was an important component of glutathione, the glutathione, which was capable of maintaining the reduced state of ubiquitin by reducing the cysteine residues within ubiquitin molecules. This enables ubiquitin to continue participating in the protein degradation process, thereby facilitating protein degradation. The presence of proline can affect protein structure, especially in the PEST sequence including proline, glutamic acid, serine, and threonine. This sequence was considered a degradation signal that can promote rapid protein degradation (Jiang et al., 2022). In addition, the ubiquitin molecule itself contains multiple lysine residues, which can serve as interfaces for the formation of polyubiquitin chains. Particularly, lysine 48 (K48) was known to be involved in targeting proteins for degradation. In the auxin metabolites, the metabolic levels of IAA-Glu (0.85), IAA-Asp (0.85), and Tryptophan (0.93) were significantly upregulated in the BSP1 group. As the level of auxin precursors metabolism increases, auxin can bind to its receptor, promoting the binding of specific target proteins (such as Aux/IAA proteins) to the E3 ubiquitin ligase complex. This leads to ubiquitination of the target protein, followed by recognition and degradation by the 26S proteasome. The genes for auxin related genes (*LOC107818819, LOC107764714. LOC107763675, LOC107774052, LOC107770412* and *LOC107775053*) were significantly up-regulated.

**Figure 9:**
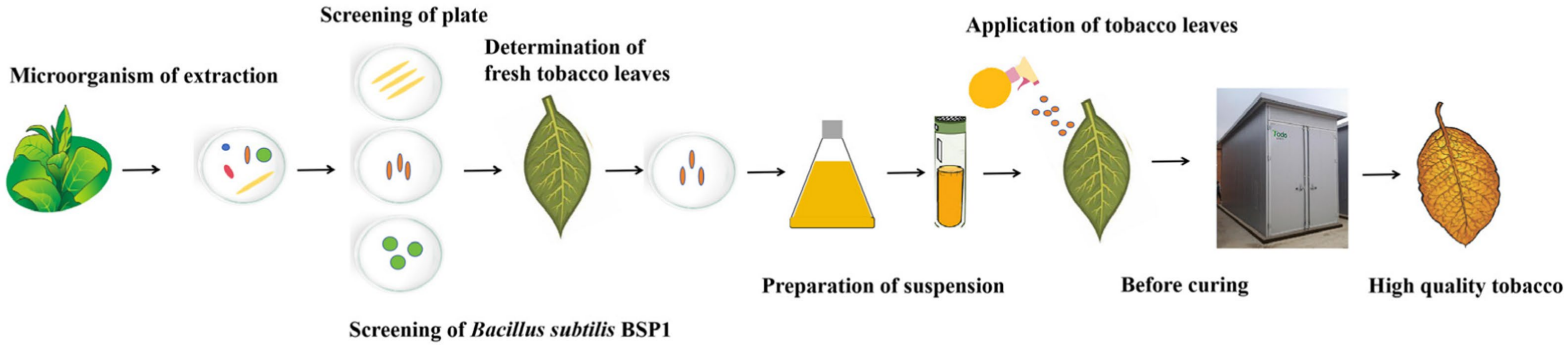
A new method to effectively degrade proteins during the curing process and to improve the quality of tobacco.

Metabolites changes were closely related to microbial community. The network association analysis was employed to investigate the interactions between microorganisms and critical metabolites (Figure 8F). This analysis demonstrated that, in comparison to the CK, the introduction of BSP1 markedly enhanced the richness and diversity of the microbial community. The microorganisms found to have strong interactions with key metabolites include groups from *Firmicutes*, *Ascomycota*, *Proteobacteria*, *Bacteroidetes*, and *Actinobacteria*. *Furthermore*, significant correlations were observed between changes in amino acid metabolites—such as L-Aspartic acid, L-Tyrosine, Tryptamine, Proline, Threonine, Tryptophan, Leucylproline, Arginine, and L-cysteine—in tobacco leaves and the presence of specific microbes like *Brevibacillus*, *Paraclostridium*, *Emergomyces*, *Bryobacter*, *Hartmannibacter*, and *Streptomyces*. The microorganisms identified from the *Firmicutes*, *Proteobacteria*, and *Actinobacteria* phyla play a crucial role in the decomposition of organic matter, thanks to their ability to produce enzymes such as cellulase and protease. Specifically, Actinobacteria and Proteobacteria were known for their efficiency in breaking down complex organic compounds into simpler ones, including amino acids (Lai et al., 2021). Among them, tryptophan and tryptamine serve as crucial precursors of auxin (Ropars et al., 2020), with their metabolic levels being up-regulated to promote auxin synthesis. Furthermore, the enhancement of auxin metabolism level facilitates the ubiquitin-protein pathway (Wei et al., 2023), thereby accelerating protein degradation. In addition, cysteine was a crucial part of the cysteine protein enzyme, and cysteine protease was believed to play a major role in the cell’s protein-degrading enzymes, thus accelerating protein degradation. Increased enzyme activity was both facilitated by the up-regulation of cysteine metabolism. The changes of nucleotide metabolism were significantly correlated with abundance of *Brevibacillus*, *Caballeronia*, *Emergomyces*, *Hymenobacter*, *Marinomonas* and *Alternaria*. These microorganisms belonged to *Firmicutes*, *Proteobacteria*. Microorganisms can break down simple N in organic matter and synthesize substances such as nucleotides and amino acids (Behera et al., 2019). And Purine metabolism and pyrimidine metabolism were the important metabolisms affecting carbohydrates (Kilstrup et al., 2005). It can promote cell energy production and accelerate protein degradation (see Figure 9).

## 4 Discussion

In this study, a new method for improving the quality of tobacco leaves was established (Figure 9). Various protein-degrading functional bacteria were successfully isolated from the surface of tobacco leaves in this study through a plate screening test, and the three most optimal functional bacteria were selected. The activity of colony proteases, as well as the protein degradation rate of fresh tobacco leaves over 24 h and the protein content over segmented 72 h, were measured. A new highly efficient protein-degrading bacterium, *B. subtilis* BSP1, was successfully identified. Additionally, for the first time, it was sprayed onto the surface of tobacco leaves before curing, and the mechanism of the functional bacteria was analyzed by combining various omics. Remarkably, a significant capability to degrade protein within tobacco leaves throughout the curing process was exhibited by this strain. Encouraging results were achieved by applying BSP1 to tobacco leaves, improving relevant substances and enzymatic activities, thereby substantially accelerating protein degradation. Detailed analyses of the metagenome, transcriptome, and metabolome of tobacco leaves under different treatments clarified the mechanisms through which BSP1 promotes protein degradation. The introduction of BSP1 protein influenced the composition and diversity of leaf microbial communities, regulating the dominance of certain bacterial groups. Notably, a shift in fungal dominance from basidiomycetes to ascomycetes was observed, facilitating the increased production of enzymes crucial for the breakdown of protein and cellulose. This shift broadly impacted tobacco leaf metabolism, including the regulation of carbon and nitrogen metabolism, as well as the metabolism of amino acids and nucleotides. Cell energy production was also enhanced, expediting the degradation of protein. Significant modifications in the concentrations of alkaloids, amino acids, and other metabolites were observed, diminishing their inhibitory effects on protein-degrading enzymes and thereby accelerating protein degradation. A significant advancement in the quest for functional bacteria in tobacco was marked by the successful isolation, identification, and functional characterization of the BSP1 strain, which shed light on its mechanisms of action. A promising approach to improving the quality of tobacco leaves during the curing process is offered by this breakthrough, leveraging the regulatory effects of microbial communities on the leaves’ biochemical composition.

An important role in plant stress tolerance was played by SOD and POD, with SOD providing protection in plant stress resistance, scavenging superoxide radicals, and mitigating the adverse effects of oxidative stress (Yan et al., 2023). Harmful substances such as hydrogen peroxide can be scavenged by POD, reducing oxidative stress, improving the plant’s ability to adapt to adversity (Yoshida et al., 2003). NR was responsible for nitrate reduction in plants, reducing nitrate to nitrite and affecting nitrogen uptake and metabolism (Campbell, 1999). MDA and PPO play crucial roles in the degradation of protein during the tobacco curing process (Wang et al., 2021). It was shown that applying a suspension of the functional bacterium *B. subtilis* strain BSP1 to tobacco leaves before the curing process markedly influenced several enzymatic activities. There was an enhancement in the activities of SOD and POD, accompanied by a decrease in MDA content. This adjustment suggests an increase in the tobacco’s resilience to stress, potentially leading to improved outcomes in the curing process by facilitating more efficient protein degradation. At the same time, there was a noticeable decrease in PPO activity, which bolstered the tobacco’s ability to withstand roasting processes and lessened the likelihood of browning reactions occurring. Moreover, an upsurge in NR activity was documented, enhancing the nitrogen metabolism within the tobacco leaves. The introduction of the BSP1 strain extended the phase of swift protein degradation, thereby facilitating a more efficient disintegration of proteins in the tobacco leaves. This process was essential for improving the quality of tobacco through the curing stage.

The microbial community composition was identified as crucial in the protein degradation process during the curing of tobacco. It was discovered that this community has the capability to modulate the chemical composition and aroma of the tobacco leaves, thereby improving their quality overall (Zheng et al., 2022). This study presented evidence that applying BSP1 during the tobacco curing process led to significant alterations in the abundance of OTUs on the leaf surface, thereby substantially affecting the microbial community composition.

In this study, transcriptome analysis was conducted to explore the mechanism behind protein degradation by the functional bacterium BSP1. Transcriptome analysis significantly enriched genes related to key enzyme activity (Supplementary Figure S1). The results showed that multiple genes related to SOD (*LOC107829594, LOC107803567, LOC107830263, LOC107800528, LOC107828097*), POD (*LOC107830366, LOC107794907, LOC107829648, LOC107813858, LOC107794678*), PPO (*LOC107802629*), NR (*LOC107823732, LOC107785409*) were up-regulated after application of BSP1. GO enrichment analysis revealed that pathways related to auxin response, organic substance response, and metabolic processes associated with hormone response were significantly enriched with DEGs following BSP1 application. In these metabolic processes, PF02519: Auxin responsive protein were up-regulated, including *LOC107768649, LOC107810690* and *LOC107811778*. This family of genes were associated with auxin response and promotes the production of auxin, which promotes protein degradation in response to the ubiquitin-proteolytic enzyme pathway (Xia et al., 2020). In addition, the gene of *LOC107829544* and *LOC107774312* was downregulated, which control storage protein synthesis. The downregulation of these genes facilitates the reduction of protein synthesis and indirectly promotes protein degradation. In addition, the functions of DEGs were estimated KEGG pathways enrichment analysis. Among them, in the metabolic pathway of nitrogen metabolism signaling pathway, the gene of *LOC107809059* and *LOC107769048* related to bifunctional monodehydroascorbate reductase were downregulated, which could Restore dehydroascorbate to ascorbic acid to induced protein synthesis (Yin et al., 2010; Mazid et al., 2011). In the metabolic pathway of tryptophan metabolism, the genes related to indole-3-pyruvate monooxygenase and L-tryptophan--pyruvate aminotransferase (*LOC107816988* and *LOC107791151*) were up-regulated, which promoted the synthesis of auxin precursors to accelerate protein degradation (Liu et al., 2012).

WGCNA was an advanced analytical approach that organizes variable genes into co-expression modules within an unsigned network, leveraging differentially expressed genes DEGs (Langfelder and Horvath, 2008). In this research, two principal modules were pinpointed for their significant associations with protein degradation processes and the metabolic actions of related metabolites. Notably, the pink module was intricately linked to the breakdown of organic macromolecules. Within this module, a collection of genes, including *LOC107807954* and *LOC107764209*, showed notable up-regulation, indicating their potential roles in enhancing the decomposition process. These genes were directly related to the ubiquitin family, and the degradation of proteins can be efficiently achieved through the ubiquitin-proteasome pathway. This might aid in the degradation of tobacco leaf proteins (Wang and Deng, 2011). Furthermore, within the green module, notable enrichment was identified in pathways related to pyrimidine and purine metabolism, alongside ATP synthesis. Specifically, genes such as *LOC107797877* and *LOC107772372* demonstrated significant upregulation. These genes were crucial for pyruvate metabolism and ATP synthesis, potentially boosting glycolytic pathways and the processes involved in energy metabolism. Such enhancements were instrumental in facilitating the efficient breakdown of protein in tobacco leaves, contributing to the overall improvement in the quality of the tobacco through enhanced metabolic functions (Börnke and Sonnewald, 2011; Santelia et al., 2015).

Significant changes were found in metabolites involved in protein degradation through metabolome analysis. For instance, the amino acid metabolite cysteine. Which was an important component of glutathione, the glutathione, which was capable of maintaining the reduced state of ubiquitin by reducing the cysteine residues within ubiquitin molecules. This enables ubiquitin to continue participating in the protein degradation process, thereby facilitating protein degradation (Turk et al., 2000). The presence of proline can influence the structure of proteins, particularly in PEST sequences (sequences rich in proline, glutamic acid, serine, and threonine). These sequences were considered degradation signals, capable of promoting rapid protein degradation (Sarfraz et al., 2021). The ubiquitin-proteasome system, lysine residues serve as the primary acceptor sites for ubiquitination. Ubiquitin forms a covalent bond with the amino group on the side chain of lysine through its C-terminal glycine. Subsequently, the protein was recognized and degraded by the 26S proteasome (Nandi et al., 2006). In addition, enhanced levels of auxin precursors lead to more efficient synthesis of auxin in tobacco leaves. Auxin can bind to its receptor, promoting the binding of specific target proteins (such as Aux/IAA proteins) to the E3 ubiquitin ligase complex. This leads to ubiquitination of the target protein, followed by recognition and degradation by the 26S proteasome (Nandi et al., 2006).

## 5 Conclusion

In this study, a new method for improving the quality of tobacco leaves was established. Various protein-degrading functional bacteria were isolated from tobacco leaves by plate test, with the three most effective strains selected. Subsequently, the activity of proteases and the protein degradation rates in tobacco leaves were assessed. *B. subtilis* BSP1, a highly efficient protein-degrading bacterium, was identified. Finally, a suspension of selected bacteria was made and sprayed on tobacco leaves before curing. Enhanced enzymatic activity, resulting in faster protein degradation during curing, was achieved through the application of BSP1 to tobacco leaves. Analyses of the microbiome, transcriptome, and metabolome revealed that protein degradation was facilitated by BSP1 through alterations in surface microbiota composition, influence on auxin synthesis and its precursor genes, and effects on the metabolism of tryptophan, cysteine, and nucleotides in tobacco leaves. Novel approaches to effectively enhance the quality of tobacco leaves were presented by the findings of this study.

## Data availability statement

The datasets presented in this study can be found in online repositories. The names of the repository/repositories and accession number(s) can be found here: the isolated strains genome can be found in the NCBI repository (https://www.ncbi.nlm.nih.gov/), accession number: PP840619.1. The raw RNA sequence reads can be found in the NCBI repository, accession number: PRJNA1117472.

## Author contributions

CJ: Conceptualization, Formal analysis, Investigation, Methodology, Software, Writing – original draft. DK: Conceptualization, Data curation, Writing – original draft. YL: Data curation, Writing – original draft. JS: Investigation, Visualization, Writing – original draft. ZC: Investigation, Visualization, Writing – original draft. MY: Resources, Supervision, Writing – original draft. SC: Resources, Supervision, Writing – original draft. CY: Resources, Supervision, Writing – original draft. ZW: Software, Validation, Writing – original draft. JJ: Software, Validation, Writing – original draft. CZ: Software, Validation, Writing – original draft. NZ: Funding acquisition, Resources, Supervision, Writing – review & editing. GS: Funding acquisition, Resources, Supervision, Writing – review & editing. QZ: Funding acquisition, Resources, Supervision, Writing – review & editing.
