# Supplementary material for: Degradation and mechanism analysis of protein macromolecules by functional bacteria in tobacco leaves
Source: Front Microbiol. 2024 Jul 5;15:1416734. doi: 10.3389/fmicb.2024.1416734 (PMC11258012; doi:10.3389/fmicb.2024.1416734)
Supplement: Supplementary file 1 [file Data_Sheet_1.docx]

Table S1 Protein degradation strains are screened

| Serial number | the diameter of the single colony degradation zone  H（mm） | Single colony diameter  C（mm） | H/C | Enzyme activity（U/mL） |
| --- | --- | --- | --- | --- |
| 1 | 3.97 | 2.47 | 1.64 | 63.61 |
| 2 | 12.8 | 5.76 | 2.21 | 65.47 |
| 3 | 15.06 | 8.11 | 1.89 | 53.94 |
| 4 | 11.54 | 4.84 | 3.18 | 66.96 |
| 5 | 6.8 | 3.86 | 2.98 | 71.05 |
| 6 | 15.8 | 7.9 | 2.27 | 52.82 |
| 7 | 7.08 | 3.69 | 1.27 | 47.24 |
| 8 | 4.04 | 3.54 | 1.14 | 49.47 |
| 9 | 14.02 | 10.16 | 1.37 | 47.61 |
| 10 | 17.34 | 6.33 | 2.87 | 36.08 |
| 11 | 11.59 | 10.26 | 1.11 | 31.62 |
| 12 | 8.16 | 3.28 | 2.51 | 56.54 |
| 13 | 9.74 | 3.81 | 2.56 | 51.32 |
| 14 | 6.66 | 3.52 | 1.9 | 52.08 |
| 15 | 8.48 | 4.42 | 2.06 | 40.16 |
| 16 | 3.28 | 2.49 | 1.31 | 34.96 |
| 17 | 8.84 | 6.67 | 1.33 | 37.2 |
| 18 | 6.58 | 3.02 | 2.74 | 74.4 |
| 19 | 8.62 | 3.02 | 2.48 | 62.48 |

Table S2 The degradation rate of tobacco leaf protein at different times

| Time | CK | T2 | T1  Degradation rate | T2 | T3 Degradation rate |
| --- | --- | --- | --- | --- | --- |
| 2h | 10.72a | 9.71a | 9.42% | 9.54a | 11.01% |
| 12h | 9.43aa | 8.34a | 11.56% | 8.47a | 10.18% |
| 24h | 8.28a | 6.65b | 22.10% | 6.81b | 17.75% |
| 36h | 8.01a | 4.76c | 40.68% | 6.13b | 23.47% |
| 48h | 7.36a | 4.51b | 41.20% | 4.55b | 40.68% |
| 72h | 7.36a | 3.77b | 48.78% | 4.1b | 44.29% |

Table S3 α diversity index of bacteria

| Constituencies | Chao1 | OTUs | Shannon | Simpson | Coverage |
| --- | --- | --- | --- | --- | --- |
| CK | 185.35±26.13 | 182.33±25.5 | 3.56±1.04 | 0.85±0.10 | 0.9996 |
| BS3 | 200.28±15.94 | 209.33±21.73* | 4.46±0.61 | 0.75±0.20 | 0.9998 |

Note: ** indicates a very significant difference (P<0.01), * indicates a significant difference (P<0.05).

Table S4 α diversity index of fungi

| Constituencies | Chao1 | OTUs | Shannon | Simpson | Coverage |
| --- | --- | --- | --- | --- | --- |
| CK | 156.52±49.34* | 153.33±46.50 | 3.73±0.22* | 0.84±0.00 | 0.9998 |
| BS3 | 96.54±5.06 | 96±5.56 | 3.05±0.30 | 0.90±0.02 | 0.9999 |

Note: ** indicates a very significant difference (P<0.01), * indicates a significant difference (P<0.05).


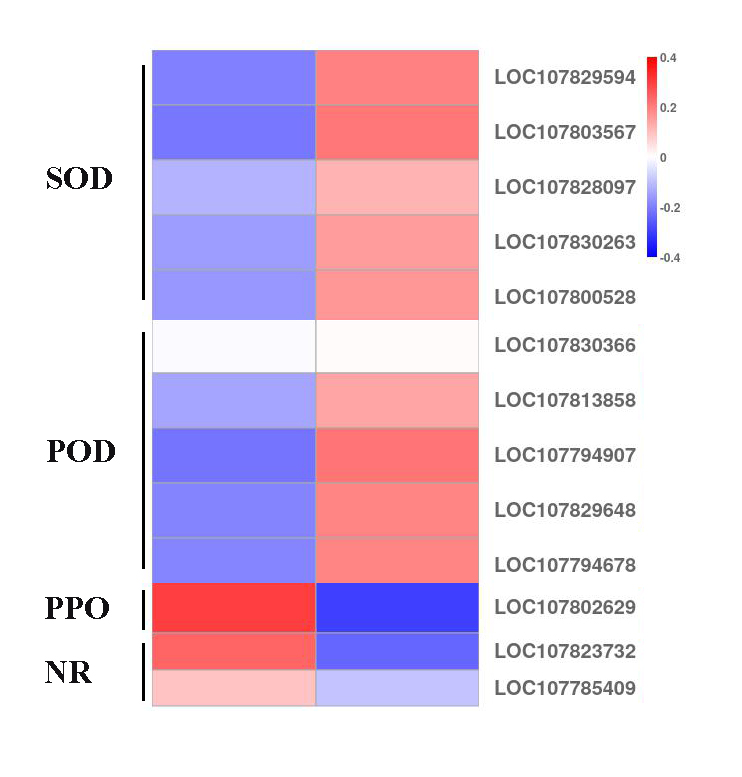


Figure S1: Key enzyme activity related genes.
